# Potential Roles of Muscle-Derived Extracellular Vesicles in Remodeling Cellular Microenvironment: Proposed Implications of the Exercise-Induced Myokine, Irisin

**DOI:** 10.3389/fcell.2021.634853

**Published:** 2021-02-05

**Authors:** Samuel Darkwah, Eun Jeong Park, Phyoe Kyawe Myint, Atsushi Ito, Michael G. Appiah, Gideon Obeng, Eiji Kawamoto, Motomu Shimaoka

**Affiliations:** ^1^Department of Molecular Pathobiology and Cell Adhesion Biology, Mie University Graduate School of Medicine, Tsu, Japan; ^2^Department of Cardiothoracic and Vascular Surgery, Mie University Graduate School of Medicine, Tsu, Japan; ^3^Department of Emergency and Disaster Medicine, Mie University Graduate School of Medicine, Tsu, Japan

**Keywords:** extracellular vesicles, muscle, integrins, myokines, tumor metastasis, tissue microenvironment, homing niche, irisin

## Abstract

Extracellular vesicles (EVs) have emerged as key players of intercellular communication and mediate crosstalk between tissues. Metastatic tumors release tumorigenic EVs, capable of pre-conditioning distal sites for organotropic metastasis. Growing evidence identifies muscle cell-derived EVs and myokines as potent mediators of cellular differentiation, proliferation, and metabolism. Muscle-derived EVs cargo myokines and other biological modulators like microRNAs, cytokines, chemokines, and prostaglandins hence, are likely to modulate the remodeling of niches in vital sites, such as liver and adipose tissues. Despite the scarcity of evidence to support a direct relationship between muscle-EVs and cancer metastasis, their indirect attribution to the regulation of niche remodeling and the establishment of pre-metastatic homing niches can be put forward. This hypothesis is supported by the role of muscle-derived EVs in findings gathered from other pathologies like inflammation and metabolic disorders. In this review, we present and discuss studies that evidently support the potential roles of muscle-derived EVs in the events of niche pre-conditioning and remodeling of metastatic tumor microenvironment. We highlight the potential contributions of the integrin-mediated interactions with an emerging myokine, irisin, to the regulation of EV-driven microenvironment remodeling in tumor metastasis. Further research into muscle-derived EVs and myokines in cancer progression is imperative and may hold promising contributions to advance our knowledge in the pathophysiology, progression and therapeutic management of metastatic cancers.

## Introduction

**Figure 1:**
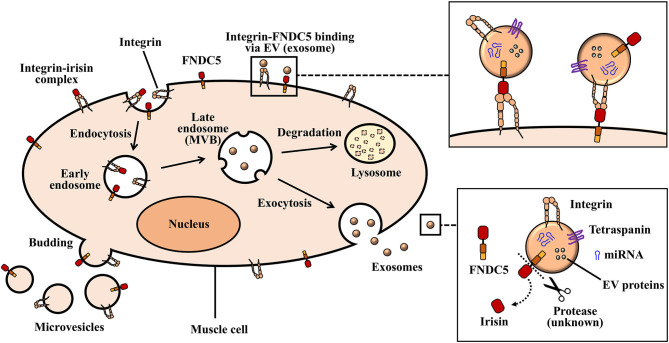
Biogenesis of exosomes and microvesicles. Inward protrusions from the plasma membrane into the cytoplasm allows for the internalization of extracellular and transmembrane proteins in the form of early endosomes. Multivesicular bodies (MVBs), as well as intraluminal vesicles originate from invaginated early endosomes. Large MVBs fuse with the plasma membrane and release exosomes into extracellular space. Vesicles that arise from the outward budding and fusion of plasma membrane make up ectosomes (microvesicles).

**Figure 2:**
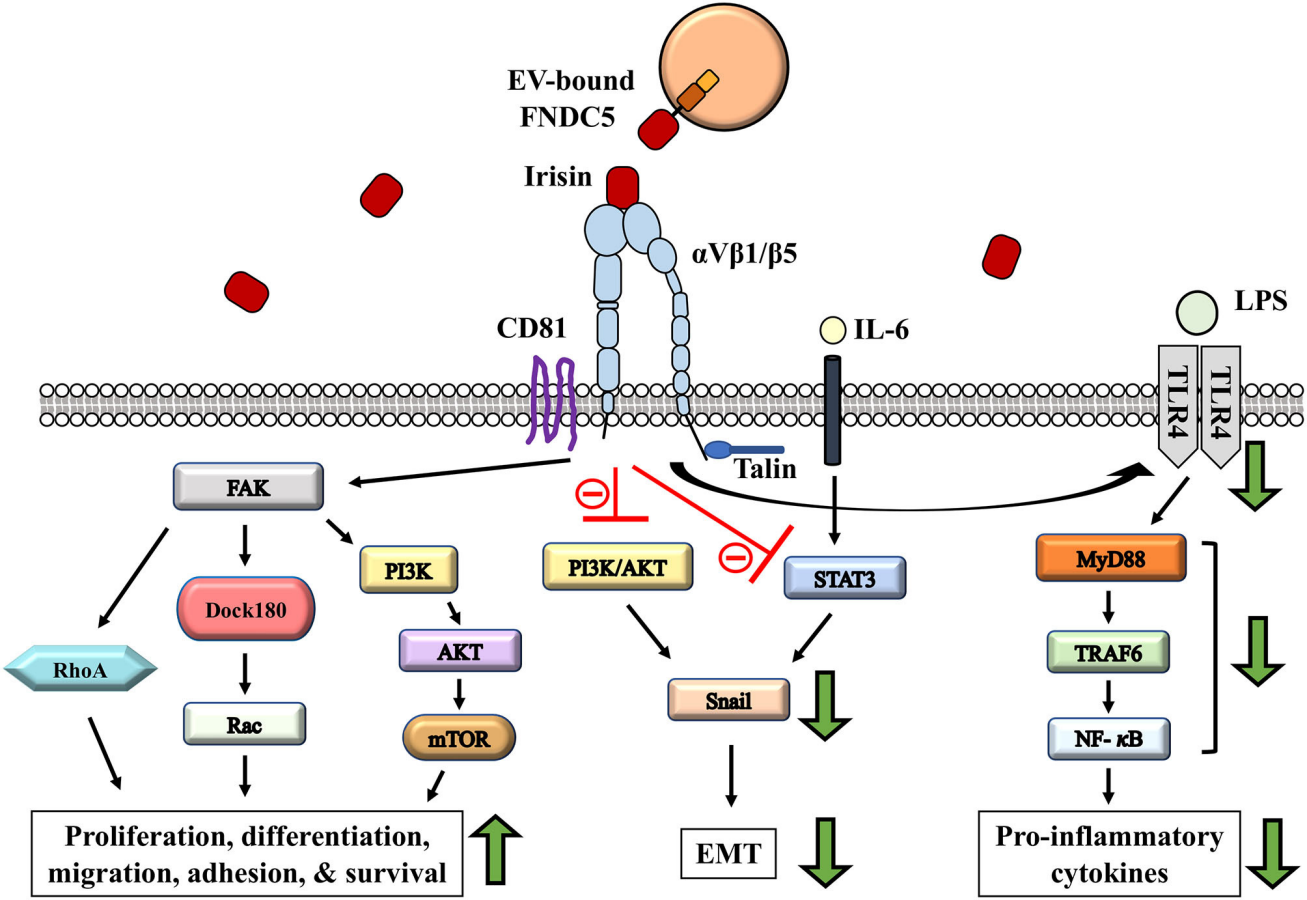
Potential role of irisin-involved integrin signaling cascade in modulating tumor microenvironment remodeling. Irisin exerts anti-inflammatory and cancer suppressing effects through the reduction in proinflammatory cytokines; via regulation of TLR4/MyD88 downstream signaling (Mazur-Bialy et al., 2017a,b). IL-6 induced EMT is regulated by irisin through STAT3/Snail signaling pathways in osteosarcoma (Kong et al., 2017). EMT inhibition is mediated by irisin signaling via the inhibition of PI3K/AKT pathway in lung cancer cells (Shao et al., 2017). Activation of CD81/integrin-mediated FAK signaling in response to irisin regulate cell survival, proliferation and migration (Oguri et al., 2020).

Extracellular vesicles (EVs) are secreted by all cell types of the body, including tumor cells and can be isolated from various biological fluids (Neven et al., 2017). Initially thought to be cellular “trash bins” containing unwanted excretes, EVs in recent times have insightfully been recognized as significant players in proximal and distal intercellular communication. A peculiar characteristic of EVs is their ability to cargo several biologic materials, capable of influencing physiological and pathological processes (Frühbeis et al., 2013; Regev-Rudzki et al., 2013; Abels and Breakefield, 2016) (Figures 1, 2). In recent times, rapid progress is being made with regards to the implementation of EVs in therapeutic interventions in the areas of inflammation, metabolic disorders, vaccination and drug delivery (Viaud et al., 2010; Lee et al., 2012; Hagiwara et al., 2014). Muscle-derived EVs have gained attention owing to their beneficial function in modulating metabolism, cell differentiation and regeneration (Choi et al., 2016; Takafuji et al., 2020a). It remains yet to be confirmed, the direct role of muscle-derived EVs in regulating cancer progression and metastasis, however, the evidence of regulatory properties of muscle EVs and myokines on other cell types hold promising cues for their potential role in tumor spread (Gannon et al., 2015; Zhang et al., 2018). In this review, we summarize the biogenesis, characteristics and functions, as well as recent findings on how muscle-derived EVs mitigate or aggravate disease conditions. We also attempt to spotlight the potential role of muscle EVs in remodeling metastatic pre-conditioning events via irisin-triggered integrin signaling.

## Biogenesis, Characteristics, and Function of Extracellular Vesicles

Extracellular vesicles (EVs) describe the heterogenous collection of a highly conserved system for intercellular communication, within which cells are able to exchange information in the form of biologically functional components: nucleic acids, proteins, and lipids (Stahl and Raposo, 2018; Wiklander et al., 2019). EVs are released by all cell types as membrane-bound spherical structures that originate from the endosome or plasma membrane, and are present in many body fluids, such as blood, urine, semen, saliva, and breast milk (Neven et al., 2017). The term, “extracellular vesicles” is generic and hence specific properties, such as the origin/source, physical characteristics and biochemical properties are required to adequately define the various subtypes (Théry et al., 2018).

Classically, EVs are broadly categorized into three vesicular types depending on their biogenesis; outward budding or fusion of multivesicular bodies (MVB) with plasma membrane. This classification gives rise to three distinct vesicle types, namely, Exosomes (EXOs), microvesicles (MVs), and apoptotic bodies (Akers et al., 2013) (Figure 1). EVs are isolated from biological samples via a myriad of isolation methods, such as precipitation, filtration, centrifugation, and a combination of different method. However, specificity and recovery of EVs may greatly vary per the isolation methods used to obtain them. Additionally, the presence of EVs in biological samples are confirmed by specific protein and lipid markers (present or absent) on the various subtypes. Well-documented guidelines outlining the recommended protocols for EVs and their documentation (provided by the International Society of Extracellular Vesicles-ISEV) exist. These guidelines encompass protocols and recommendations designed for the efficient characterization, isolation, and functional studies of EVs (Théry et al., 2018).

### Exosomes

Exosomes, which are nano-sized vesicles ranging between small diameters of about 30–150 nm, are derived from the endosomal system. The formation and secretion of exosomes begin with the invagination of the plasma membrane (endocytosis) to form a cup-like structure harboring cell-surface proteins and extracellular deposits (Kalluri and LeBleu, 2020). This invagination forms an early endosome which then mature to form late endosomes. Subsequently, intraluminal vesicles (ILVs) are formed by the progressive, inward budding of the late endosomal membrane. The accumulation of ILVs in the late endosome generates multivesicular bodies (MVBs) (Kalluri and LeBleu, 2020; Yue et al., 2020). At this stage, MVBs can fuse with autophagosomes or lysosomes for the degradation of their endosomal contents. Degraded components can then be recycled by the cell. Alternatively, MVBs (containing mature or late endosomes) that do not traffic to lysosomes move toward the cytoplasmic side of the plasma membrane with the aid of the cellular cytoskeletal and microtubule network (Kalluri and LeBleu, 2020). Here, MVBs are able to dock via MVB-docking proteins and fuse with the plasma membrane as exocytic MVBs. Exocytic MVBs release their lipid bi-layered ILV contents to the extracellular space as exosomes (Kalra et al., 2016) (Figure 1).

It still remains to be completely understood, the processes that govern the formation of ILVs in MVBs and their further release as exosomes. However, a proposed mechanism implicated in this process involves the Endosomal Sorting Complex Required for Transport (ESCRT). ESCRTs comprise of several proteins that assemble into four ESCRT complexes: namely ESCRT-0, -I, -II, and -III, in association with others like ALIX and vacuolar protein sorting-associated proteins (VPS4, VTA 1) (Colombo et al., 2013). Initiating this process is the organization of endosomal membranes into specialized units that are highly enriched for the tetraspanin class of membrane proteins. Tetraspanins (e.g., CD63, CD9, and CD81) function to cluster the required proteins for ILV formation and are considered common markers for the identification of exosomal vesicles (Pols and Klumperman, 2009). ESCRTs are then recruited to the site of ILV formation where ESCRT-0 recognizes and binds to ubiquitinated proteins present on the outside of the endosomal membrane. ESCRTs-I and -II are then assembled to the cytosolic area to initiate and drive intraluminal membrane budding. The assembling of ESCRT-I/II is reportedly stimulated by some factors, such as the abundance of phosphatidylinositol 3-phosphate (PIP3) in the membrane early endosomes, ESCRT-0 proteins (e.g., hepatocyte growth factor-regulated tyrosine kinase substrate-HRS) and the ubiquitination of the cytosolic tail of endocytosed proteins and/or their curved membrane topology (Henne et al., 2013; Abels and Breakefield, 2016). Upon activation of ESCRT-II, ESCRT-III is recruited through an associated protein, ALIX. ALIX binds to the tumor susceptibility gene 101 (TSG101) component of ESCRT-I complex and also to the charged MVB protein 4A (CHMP4A) components of ESCRT-III, thus, serving as an intermediate between ESCRT-III and ESCRT-I association (McCullough et al., 2008). ESCRT-III in conjunction with deubiquitinating enzymes then finalize the processes that involve vesicle closure and the detachment of ILVs from the membrane. ESCRT-III forms structures that constrict budding neck followed by scissoring of the membrane (driven by accessory proteins, such as VPS4) and finally, the dissociation and recycling of the ESCRT machinery (Kalra et al., 2016; Schöneberg et al., 2017).

Recent studies identify an alternate ESCRT-independent pathway for ILV formation which involves clustering of sphingomyelin in lipid rafts where it is converted to ceramide by sphingomyelinases. The accumulation of ceramide subsequently induces the merging of microdomain, thus, triggering ILV formation (Stuffers et al., 2009; Kalra et al., 2016). As such, exosomes presumed to be formed through this pathway may lack the expression of common markers, such as ALIX and TSG 101 involved in the endosomal pathway. Small RAB GTPases facilitate the docking, fusion and secretion of exosomes. For example, RAB27A promotes docking of MVBs and fusion to the plasma membrane, whiles RAB27B is involved in vesicle transfer from the Golgi to MVBs, and mobilization of MVBs to the actin-rich cortex under the plasma membrane (Ostrowski et al., 2010; Kalra et al., 2016).

### Microvesicles

Vesicles that arise through direct outward budding and fission of the plasma membrane are known as microvesicles (MVs) or ectosomes (Figure 1). These range between relatively larger diameters of about 50–2,000 nm (Akers et al., 2013). Ectosome biogenesis involves plasma membrane phospholipid re-arrangements/redistribution, as well as, contractions of cytoskeletal proteins.

Enzymes (collectively described as aminophospholipid translocases) involved in the exchange of lipids between the inner and outer leaflet of the cell membrane for asymmetric maintenance are activated to induce changes within the bilayer to allow for budding and membrane abscission. Flippases are translocases required for the transfer of phospholipids from the outer leaflet to the inner leaflet and floppases, on the other hand, transfer phospholipids from within out. Following this, actin and myosin interactions within the cytosol induce the contraction of cytoskeletal structures; a mechanism that underlies bud formation and subsequent release of ectosome portions. Microvesicles, like exosomes, can be highly enriched in sets of proteins and are particularly identified with markers, such as vesicle-associated membrane protein 3 (VAMP3) and ADP-ribosylation factor 6 (ARF6) (Hugel et al., 2005; Muralidharan-Chari et al., 2009).

### Apoptotic Bodies

During programmed cell death, apoptotic cells undergo several stages. These stages begin with condensation of the nuclear chromatin and then membrane blebbing. Cellular components that undergo disintegration are then processed into distinct membrane-enclosed vesicles. These vesicles comprise of a type of EV known as apoptotic bodies that range in sizes between 500 and 4,000 nm. Apoptotic bodies pack organelles or cytosolic components and are capable of transferring genetic information from cell to cell via their uptake (Bergsmedh et al., 2001; Cocucci et al., 2009; Kalra et al., 2016).

## Cellular Microenvironments (Niches) and Extracellular Vesicles

Accumulating evidence has revealed that EVs are involved in the inter-cellular communication of a wide range of physiology and pathophysiology (Park et al., 2019a; Wortzel et al., 2019; Kalluri and LeBleu, 2020). In the following sections, we focus on an emerging topic (Myint et al., 2020): the roles of EVs in the regulations of microenvironments supporting the trafficking of normal (lymphocytes) and transformed (e.g., cancer) cells in sections ‘Cellular Microenvironments (Niches) and Extracellular Vesicles' and ‘EV-Mediated Remodeling of Cancer Microenvironment', respectively.

Efficient migration and retention of specific cell types in various compartments of the body are supported by a special microenvironment (homing niche). Such microenvironments have the ability to maintain specific cell populations away from apoptotic and differentiation signals, as well as provide an ambience (cellular structures, extracellular proteins and soluble factors) for proliferation (Moore and Lemischka, 2006).

### EVs in Lymphocyte Homing

The regulated migration of lymphocytes is necessary for their effective function. This regulation phenomenon termed, “lymphocyte homing” enables for the trafficking of lymphocytes to specific sites of the body. Lymphocyte homing has been particularly demonstrated in gut immunology. Antigen-primed naïve T lymphocytes in the gut lymph nodes are imprinted to allow them home back into gut tissues as effector or memory T lymphocytes: a necessity for efficient adaptive immunity in the gut mucosa (Myint et al., 2020). High endothelial venules (HEV) of the gut associated lymphoid tissues (GALT) highly express mucosal addressin cell adhesion molecule-1 (MAdCAM-1) that engages the receptor integrin α4β7 expressed on activated lymphocytes. This interaction enables for the homing of antigen-specific B and T lymphocytes into gut tissues (Streeter et al., 1988; Berlin et al., 1995). The inability of β7 integrin deficient mice to mount antigen-mediated humoral responses in the gut affirms the critical role of integrin α4β7 in gut mucosa B-cell immune responses (Schippers et al., 2012). Additionally, HEVs in GALT chemo-attract chemokine receptor 7 (CCR7)-bearing naïve and central memory T lymphocytes via CCL21 (Okada et al., 2002). Gut-tropic lymphocytes expressing chemokine receptor 9 (CCR9) are signaled by CCL25 and integrin α4β7/MADCAM-1 expressing epithelial cells of HEV to facilitate their migration and homing to the gut (Myint et al., 2020). Lymphocyte homing niche in the gut is physiologically regulated. The expression of MAdCAM-1 in the gut is stabilized in limited levels to maintain homeostasis (Pabst et al., 2000). Elevated signal from inflammatory mediators as seen in gut inflammatory diseases upregulate MAdCAM-1 expression and promote the accumulation of activated T lymphocytes that abundantly express integrin α4β7 (Myint et al., 2020).

Remodeling of homing niches to regulate the tissue-specific migration and recruitment of activated lymphocytes has been demonstrated in recent studies. One mechanism by which the homing niche is remodeled appears to be through gut-tropic derived exosomal secretions, demonstrated by Park et al. (2019b). Activated gut-tropic T lymphocytes secrete exosomes that highly express functional integrin α4β7 with the ability to bind MAdCAM-1 and preferentially home to the gut mucosa. These α4β7 expressing exosomes function to suppress MAdCAM-1 through the cargo and delivery of microRNA milieu that target the transcriptional factor, Nirenberg-Kim (NK) 2 homeobox 3 (NKX2.3) necessary for MAdCAM-1 regulation. Consequently, other homing niche factors, such as the chemokines CCL25 and CCL28 were suppressed by gut-tropic lymphocyte-derived exosomes (Park et al., 2019b). It is therefore not far-fetched to consider exosomes of lymphocytes and other host cells as key remodeling regulators of homing niches to maintain homeostasis.

### Other Homing Niches

EV-mediated regulation of homing niches (by modifying the expression of adhesion molecules and chemokines) may operate in stem cell trafficking, and warrants further research. Here we explain the overview of how integrins and chemokines govern stem cell trafficking. Physiologically, stem cells home to specific tissue areas via chemoattraction. For example, hematopoietic stem cell precursors home to the bone marrow after selectin- mediated braking (E-selectins, P-selectins, and L-selectins) that facilitates migration on adhesion ligands expressed by vascular endothelium (Liesveld et al., 2020). Following braking, intracellular signaling cascades are activated through chemokine- chemokine receptor interactions (e.g., CXCL12/CXCR4) to trigger the activation states of integrins like α_L_β_2_ (LFA-1) and α_4_β_1_ (VLA-4). This facilitates the arrest of cells and their subsequent migration via integrin/cell adhesion molecule (CAM) interaction (Schweitzer et al., 1996; Huttenlocher and Horwitz, 2011; Sahin and Buitenhuis, 2012). Increased CXCL12/CXCR4 signaling is implicated in the retention of leukocytes, such as mature neutrophils at inflammatory sites (demonstrated in tissue-damaging inflammatory diseases) (Yamada et al., 2011; Isles et al., 2019), or in the bone marrow (demonstrated in Warts, Hypogammaglobulinemia, Infections, and Myelokathexis (WHIM) syndrome-associated neutropenia) (Kawai and Malech, 2009). Additionally, FoxP3^+^regulatory T cells trafficking and homing to hematopoietic stem cell niche in the bone marrow involves the signaling of CXCR4 (Zou et al., 2004; Hirata et al., 2018). It has been demonstrated that, loss of adhesion molecules like integrins (e.g., β7-integrins: α4β7, αEβ7) and selectins (e.g., P-selectin) influences stem cell and lymphocyte trafficking (Frenette et al., 1996; Schön et al., 1999; Lucas, 2019).

The subventricular zone (SVZ) of the brain is another example of a host site that serves as a niche for neural stem cells (NSC), with inflammation playing a key role in the homing and recruitment of NSC for CNS regeneration. Increased expression of stromal derived factor 1α (SDF-1α or CXCL12) by astrocytes and endothelial cells of ischemic areas in the brain, as well as their constitutive expression and activation of CXCR4 (receptor of CXCL12), facilitate NSC migration toward ischemic brain explants (Imitola et al., 2004). LeX-positive NSCs (denoting a subpopulation of SVZ adult stem cells that express the extracellular matrix (ECM)-associated carbohydrate, Lewis X) potentially differentiate into cells with neuronal phenotypes following transplantation. These cells acquire enhanced migratory and proliferative characteristics upon exposure to SDF-1α (Corti et al., 2005). Other soluble factors involved in the migration and recruitment of NSCs include vascular endothelial growth factor (VEGF), interleukin-6, hepatocyte growth factor (HGF) and platelet derived growth factor (PDGF) (Garzón-Muvdi and Quiñones-Hinojosa, 2009). Just as with the effect of soluble growth factors on NSC homing, the expression of integrins, such as α6β1 in SVZ neurogenic niche play a vital role in NSC recruitment, demonstrated in a rodent model of cerebral ischemia (Prestoz et al., 2001).

## EV-Mediated Remodeling of Cancer Microenvironment

Cancer cells secrete exosomes that remodel homing niches to promote migration, retention and proliferation of tumor cell and/or other cell types, thus, tumor exosomes form a part of the tumor-derived factors necessary for pro-tumor microenvironment formation and metastatic niche pre-conditioning (Hoshino et al., 2015). Exosomes released by breast cancer cells are delivered to distal cells of target organs, such as the lung and liver, where they induce the expression of proinflammatory genes through the activation of Src-kinase signaling. Thus, the initiation of exosome-mediated chronic inflammation contributes to the remodeling of distal tissues to favor tumor metastasis. Concomitantly, breast cancer exosomes highly expressing integrins αvβ5 and α6β4 distribute to the fibronectin-enriched ECM of the liver and laminin-enriched ECM of the lung, respectively. As a result, tissues within which these integrin-directed exosomes are specifically distributed become prone to inflammatory-mediated remodeling to support the cultivation of pre-metastatic niche and consequently, predisposed to metastatic cancers (Hoshino et al., 2015; Myint et al., 2020).

Alluring to the evidence that non-tumor cells and their exosomes function to tightly regulate trafficking and homing to specific niches for regeneration, repair and homeostasis (Prestoz et al., 2001; Park et al., 2019b), cancer cells and cancer-derived exosomes on the other hand play a contributory role in remodeling niches to support cancer metastasis (via chronic inflammation, angiogenesis, increased vascular permeability, etc.) (Hoshino et al., 2015; Myint et al., 2020).

## Muscle–Derived EVs and Myokines in Microenvironment Remodeling

Growing evidence suggests that major risk factors of cancer development and progression include metabolic disorders that facilitate chronic inflammation, thus, metabolic syndrome is consistently associated with increased risk of common cancers (Esposito et al., 2012). Although mechanisms underlying the link between metabolic syndrome and cancer risk are not fully understood, metabolic syndrome represents a proxy marker for several risk factors, such as lack of physical activity, high dietary fat intake and oxidative stress (Alberti et al., 2009). Adipocytes and infiltrating immune cells induce a systemic low-grade chronic state of inflammation by producing inflammatory mediators/immune cell chemo-attractants (interleukin-1β [IL-lβ], interleukin-6 [IL-6], tumor necrosis factor-α [TNF-α], and monocyte chemoattractant protein-1[MCP-1]) and increasing the circulation of free fatty acids to promote the establishment of tumorigenic microenvironment. This is evident in excess adiposity, insulin resistance, aberrant glucose metabolism and particularly, central obesity (visceral adiposity) (Harvey et al., 2011). Hyperglycemia, a hallmark of metabolic syndrome, is implicated in the dysregulation of growth factors and metabolic hormones like insulin and Insulin-like growth factor (IGF-1). Hyperglycemia-induced suppression of IGF-binding protein synthesis and the promotion of IGF-1 synthesis increase the amounts of bioavailable IGF-1 in circulation; an established critical risk factor for several malignancies (Pollak, 2008). Downstream signaling pathways of activated receptor tyrosine kinases, such as IGF-1 receptor activate the Akt cascades which are commonly altered in epithelial cancers. Typical in tumors and tissues of diabetic rats, is the increased activation of downstream mediators, such as mammalian target of rapamycin (mTOR) (Wong et al., 2010; De Angel et al., 2013).

The association between obesity and physical inactivity has been shown in several studies (Gustat et al., 2002; Golbidi et al., 2012). Evidently, exercise and muscle training prove to be beneficial in the prevention and management of obesity or other related metabolic distress. Decreased daily physical activity in healthy individuals is linked to undesired consequences in metabolism, such as reduced insulin sensitivity and elevated adiposity (Olsen et al., 2008). Exercise induces anti-inflammatory effects, boosts antioxidant capacity, and regulates glucose/fat metabolism. Skeletal muscle activity has been found to be beneficial in regulating metabolism and inflammation within several tissues, including adipose tissues. Contracting muscles release myokines, such as interleukin 6 (IL-6) that positively impact inflammation. IL-6 acts as a pro-inflammatory and an anti-inflammatory mediator, however, studies have shown that exercise-induced muscle-derived IL-6 exerts anti-inflammatory effects. The anti-inflammatory property of IL-6 is shown by way of an inhibitory effect on TNF-α, IL-10, and IL-1β, to protect against TNF-induced insulin resistance (Festa et al., 2000; Febbraio and Pedersen, 2005). Myokines secreted by muscle tissues into the extracellular space are capable of modulating homeostasis in the bone, pancreas and adipose tissues. Such myokines like IL-6, irisin, myostatin, and interleukin-15 (IL-15) play a key role in the crosstalk between muscle and adipose tissues (Leal et al., 2018). Recent articles, such as that of Mika et al., review the potential benefits of exercise-induced release of myokines via the alterations in adipose tissue fatty acid metabolism (e.g., increased lipolysis and reduced fatty acid uptake). Chronic exercise impacts the profile of adipokines released from adipose tissues, as well as promote the “beiging” of adipocytes (Mika et al., 2019).

Intriguingly, the provision of beneficial myokines is not confined to muscle cells. Muscle-derived extracellular vesicles (EVs) cargo and distribute myokines, muscle specific microRNAs (myomiRs) and other soluble factors to proximal and/or distal tissues, thus, crosstalk between muscle and other tissues is at least in part, mediated by EVs (Whitham et al., 2018), from muscles. Exercise and physical activity have been demonstrated to augment the release of muscle EVs into circulation.

### Skeletal Muscle EVs

Both myoblasts and non-proliferating myotube forms of skeletal tissue are capable of secreting EVs, *in vitro* (Forterre et al., 2014a; Choi et al., 2016). Skeletal muscle-derived EVs have been demonstrated to be key players in muscle physiology and systemic homeostasis via paracrine actions (Rome et al., 2019). The release of small EVs into circulation is particularly enhanced by physical exercise, inflammation/stress, and several muscle-related conditions (Frühbeis et al., 2015; Barone et al., 2016). Skeletal muscle is a major contributor of exercise-induced secreted molecules. Some of these secreted molecules were identified in EVs harvested from conditioned medium (CM) of myotube cultures (Forterre et al., 2014b; Deshmukh et al., 2015). Skeletal muscle-derived EVs play vital roles in the differentiation and regeneration of muscle tissue by triggering cues for myogenic processes and myofiber regeneration. Exosomes derived from human skeletal myoblasts were found to contain various myogenic factors, such as insulin-like growth factors (IGFs), fibroblast growth factor-2 (FGF2), and hepatocyte growth factor (HGF) that enhanced terminal myogenic differentiation of stem cells, *in vitro* (Choi et al., 2016). Conversely, skeletal muscle-derived exosomes may be implicated in suppressing myogenic events. During inflammatory conditions or tissue damage, inflammatory cells and the milieu of pro-inflammatory mediators, such as chemokine ligand 2 (CCL2) induce the production of muscle EVs which package more myostatin (negative regulator of myoblast proliferation and differentiation) and less decorin (myostatin antagonist) (Tidball, 2017; Kim S. et al., 2018).

Crosstalk between the skeletal system and the peripheral nervous system have been demonstrated to be at least in part, mediated by skeletal muscle-derived exosomes. Motor neuron regeneration and survival has been found to be positively impacted by EVs released from muscle cells, *in vitro* (Madison et al., 2014). Additionally, skeletal muscle denervation induced the release of muscle EVs that preferentially support motor neuron regeneration accuracy. Muscle-derived EVs (containing muscle specific markers, such as α-sarcoglycan) were taken up by denervated, but not naïve nerve tissue around the neuro-muscular junction and significantly induced the exclusive projection of motor neurons to muscle branch, thus, ensuring anatomically accurate motor neuron regeneration in rats (Madison and Robinson, 2019). However, mechanisms underlying exactly how muscle-derived EVs are taken up and localized within the denervated nerve remain a knowledge gap incompletely bridged.

MicroRNAs, carried by muscle EVs have been implicated in the crosstalk between muscle and other cell types (Jalabert et al., 2016; Nie et al., 2019), particularly between muscle and bone in the events of bone remodeling. Skeletal muscle-derived exosomes enriched in miR-27a-3p positively impacted osteogenesis by delivering and increasing miR-27a-3p to target adenomatous polyposis coli (APC), thereby, activating the Wnt/β-catenin pathway to promote osteogenic differentiation of MC3T3-E1 pre-osteoblasts (Xu Q. et al., 2018). Another area of evidence for muscle-bone crosstalk mediated by muscle EVs is osteoclast biogenesis in osteoporosis. Mouse muscle cell line (C2C12 myoblasts)-derived EVs suppressed osteoclast formation and mitochondrial energy metabolism in bone marrow cells (Takafuji et al., 2020a). This group demonstrated that Myo-EVs were successfully taken up by bone cells in culture and suppressed the expression of RANKL-induced osteogenic factors, such as cathepsin K (CTSK), nuclear factor of activated T-cells 1 (NFATc1) and dendrocyte expressed seven transmembrane protein (DCSTAMP). Evidently, Myo-EVs suppressed the oxygen consumption rate and expressions of peroxisome proliferator-activated receptor co-activator 1 beta (PGC1β), NADH-ubiquinone oxidoreductase chain 4 (ND4) and cytochrome c in mouse bone cells (Takafuji et al., 2020a), possibly by an miRNA-mediated inhibition of CREB/PGC1β pathway in osteoclast precursors (Takafuji et al., 2020b). Oxidative stress has been shown to modify miRNA contents of Myo-EVs; miR-34a is elevated in C2C12 derived EVs following oxidative stress. Muscle EVs enriched in miR-34a home to the bone and induce senescence of bone marrow-derived stem cells by decreasing Sirtuin1 expression on primary bone marrow cells (Fulzele et al., 2019).

In metabolic abnormalities like diabetes and insulin resistance, paracrine and/or endocrine effects of Myo-EVs have been reported. A study revealed that the pancreas takes up muscle EVs *in vivo*. Such EVs transferred muscle specific miRNAs to pancreatic beta cells and islets, thereby, modulating gene expression and proliferation (demonstrated *in vitro* with MIN6B1 pancreatic cell line). In this study, muscle EVs derived from insulin resistant skeletal muscle were particularly enriched in miR-16 cargo that could be transferred to pancreatic cells and regulate PTCH1, potentially contributing to the pathogenesis of type-2 diabetes (Jalabert et al., 2016).

As positive regulators of metabolic function, exercise-induced skeletal muscle exosomes potentially promote adipocyte lipolysis. IL-15 is known to be highly expressed in skeletal muscle, and acts to decrease fat depots in adipose tissue (Quinn et al., 2005). Additionally, myonectin, a recently established myokine involved in lipid metabolism has been identified as a link between skeletal muscle and systemic lipid metabolism (Seldin and Wong, 2012). Considering the abundance of IL-15 mRNA in the contracting skeletal muscle (Nielsen et al., 2007) and the exercise-induced release of muscle IL-6 in vesicular structures (Lauritzen et al., 2013), it is not far-fetched to speculate the packaging and cargo of this lipolysis-influencing myokine in skeletal muscle derived exosomes. Alterations in skeletal muscle (e.g., muscle activity and injury) have been shown to influence the release of key microRNAs (e.g., miR-21, miR-148b, and miR-486 in tissues, plasma and exosomes) that regulate insulin responsiveness (D'Souza et al., 2018).

### Cardiomyocyte Derived EVs

Although not classically described as typical secretory cells, cardiac muscle cells can be induced to release EVs, *in vitro* (Gupta and Knowlton, 2007; Chistiakov et al., 2016). Cardiomyocytes, which constitute the prime contractile cell type in cardiac tissue, display augmented secretions of EVs under several cellular stress conditions like inflammation, injury, hypoxia, alcohol exposure and glucose starvation (Garcia et al., 2015; Yu and Wang, 2019). Similar to skeletal muscles, cardiac muscle EVs cargo nucleic acids, including miRNAs that can regulate gene expression in recipient cells (Waldenström et al., 2012).

Oxygen and nutrient supply are imperative for the efficient function of the myocardium. As such, blood vessels feeding the myocardium play a key role in cardiac function. The crosstalk between cardiac myocytes and intra-cardiac endothelial cells has been established. This crosstalk is vital to meet the metabolic needs for efficient heart function (Garcia et al., 2016) and has been found to be mediated by paracrine signal molecules, direct cell-cell communication, and EVs (Colliva et al., 2020). In the light of cardiomyocyte-endothelial cell communication, cardiomyocyte-derived EVs effect both anti-angiogenic and pro-angiogenic regulatory functions (Ribeiro et al., 2013; Wang et al., 2014; Chistiakov et al., 2016). Heat shock proteins (Hsps) well-known to protect cardiomyocytes from stresses (Willis and Patterson, 2010; Edwards et al., 2011; Yu and Wang, 2019; Yu et al., 2019) are highly expressed in muscle cells and secreted from cardiomyocytes via exosomes. Particularly, cardiomyocyte Hsp20 released through exosomes by the non-classical endoplasmic pathway promote angiogenesis; enhanced proliferation, migration, and tube formation of endothelial cells by Hsp20 via the activation of vascular endothelial growth factor receptor (VEGFR) signaling cascade (Zhang et al., 2012). The crucial role of cardiac microvascular endothelial (CME) cells in protecting against myocardial injury by way of activating endothelial nitric oxide synthase (eNOS) has been evidently linked to signals from cardiomyocytes. A study reported that, cardiomyocytes regulated CME cells via EV transfer of long intergenic non-protein coding RNA, Regulator of Reprogramming (Linc-ROR) to target miR-145-5p, ultimately resulting in the activation of eNOS pathway (Chen et al., 2020). Conversely, anti-angiogenic pathways are mediated by cardiomyocyte-derived EVs and contribute to endothelial dysfunction, insufficient myocardial angiogenesis and cardiovascular complications (Wang et al., 2014). Myocyte-derived exosomes from type-2 diabetic rats but not wild type inhibited proliferation, migration, and tube formation of cardiac endothelial cells in culture through miRNA regulation. It was observed that diabetes induced the release and transfer of exosomes harboring high levels of miR-320 (while being less enriched in miR-126) that target angiogenic factors (Hsp20 and insulin-like growth factor-1) in endothelial cells (Wang et al., 2009, 2014), potentially contributing to diabetes-related vascular dysfunction.

Cardiac remodeling is crucial in the pathophysiology of cardiac injury and hence, should be appropriately regulated to prevent irreversible changes to the structure and function of the myocardium (Tracy et al., 2020). Cardiac muscle exosomes play a key role in regulating myocardial remodeling by the transfer of encapsulated miRNAs to target fibrotic factors (Chaturvedi et al., 2015; Chistiakov et al., 2016). A study revealed that miR-133a from cardiomyocyte exosomes targeted type IA1 collagen (Col1A1) and connective tissue growth factor, resulting in decreased myocardial fibrosis in hypertensive rats (Castoldi et al., 2012). Other cardiomyocyte-derived exosome miRNAs that have been identified to promote fibrosis and fibroblast differentiation in animal models include miR-208a (Yang et al., 2018), miR-92a (Wang et al., 2020) and miR-195 (Morelli et al., 2019). While a number of exosome-derived miRNAs contribute to myocardial fibrosis, others, such as miR-378 tend to exhibit a protective effect by targeting mitogen-activated protein kinase kinase 6 (MKK6) to suppress p38 mitogen-activated protein kinase phosphorylation in cardiac fibroblasts (Yuan et al., 2018). Other miRNAs implicated in regulating the fibrotic cascades in this regard include miR-29b (Chaturvedi et al., 2015) and miR-373 (Xuan et al., 2019).

Recently, the roles of cardiomyocyte-derived exosomes have expanded to encompass a role in mechanisms that underlie the therapeutic efficacy of stem cells in myocardial infarction (Hu et al., 2018). Cardiomyocyte exosomes obtained from culture conditioned medium under stress microenvironment (peroxide-induced oxidative stress) hastened stress-induced injury of bone marrow-derived stem cells (Hu et al., 2018).

### Smooth Muscle–Derived EVs

Vascular smooth muscles in a synthetic or proliferative, non-contractile state exhibit an increased production of extracellular vesicles (Kapustin and Shanahan, 2016; Schurgers et al., 2018), demonstrated by the inverse relationship between the expression of contractile vascular smooth muscle cell markers and exosome secretion (Kapustin et al., 2015). Inflammatory cytokines and growth factors like tumor necrosis factor-α (TNF-α) and platelet-derived growth factor (PDGF), respectively stimulate the phenotypic transition and exosome secretion by vascular smooth muscle cells (Kapustin and Shanahan, 2016). The activation of vascular smooth muscle cells (SMC) form one of the key events in atherogenesis and the onset of vascular complications, such as stroke and myocardial infarction (Bennett et al., 2016). Overexpression of Krüppel-like factor 5 (KLF5), a transcription factor requisite for mediating SMC proliferation and migration in vascular remodeling events, mediates the secretion of exosomes enriched on miR-155 from human smooth muscle cells. The transfer and uptake of SMC-derived exosomal miR-155 by endothelial cells have been shown to impair endothelial integrity by suppressing their proliferation, migration, tube formation (angiogenesis), and expression of tight junction proteins (Zheng et al., 2017).

Recently, smooth muscles of the pulmonary vasculature have been shown to release EVs that package a myriad of RNA transcripts transferrable to pulmonary arterial endothelial cells (de la Cuesta et al., 2019). Migration and apoptosis of pulmonary arterial smooth muscle cells account for the modulation of miR-143. As a result, exosomes derived from these cells selectively pack abundant miR-143-3p that exhibit pro-angiogenic and pro-migratory effects on recipient pulmonary arterial epithelial cells (Deng et al., 2015). In another recent finding, pulmonary arterial epithelial cells cultured with exosomes derived from platelet-derived growth factor-stimulated smooth muscle cells showed an enhanced migratory but not proliferative ability via the effect of exosomal miRNAs. It was observed that exosomes from PDGF-stimulated smooth muscle cells were deficient in miR-182, miR-1246, and miR-486 and this alteration proved critical for the enhanced migratory phenotype of endothelial cells in pathologic states (Heo et al., 2020).

The microenvironment or matrix of the vasculature is to a large extent regulated by microvesicles secreted from muscle cells, endothelial cells, and infiltrating immune cells of blood vessels (Reynolds et al., 2004; New et al., 2013). Small EVs secreted by vascular smooth muscle cells normally cargo various factors like the high affinity calcium ion-binding matrix Gla protein (MGP) and fetuin-A that function to inhibit calcification, thus protecting against vascular mineralization and the formation of atherosclerotic plagues (Reynolds et al., 2005). However, inflammation and mineral imbalance influence this protective role by inducing the release of EVs enriched in calcification promoters and depleted of inhibitors, thus, conditioning an environment conducive for vascular mineralization and stiffening (Reynolds et al., 2005; Kapustin et al., 2011).

## Emergence of Irisin Signaling and Potential Implications for Microenvironment Remodeling

**Figure 3:**
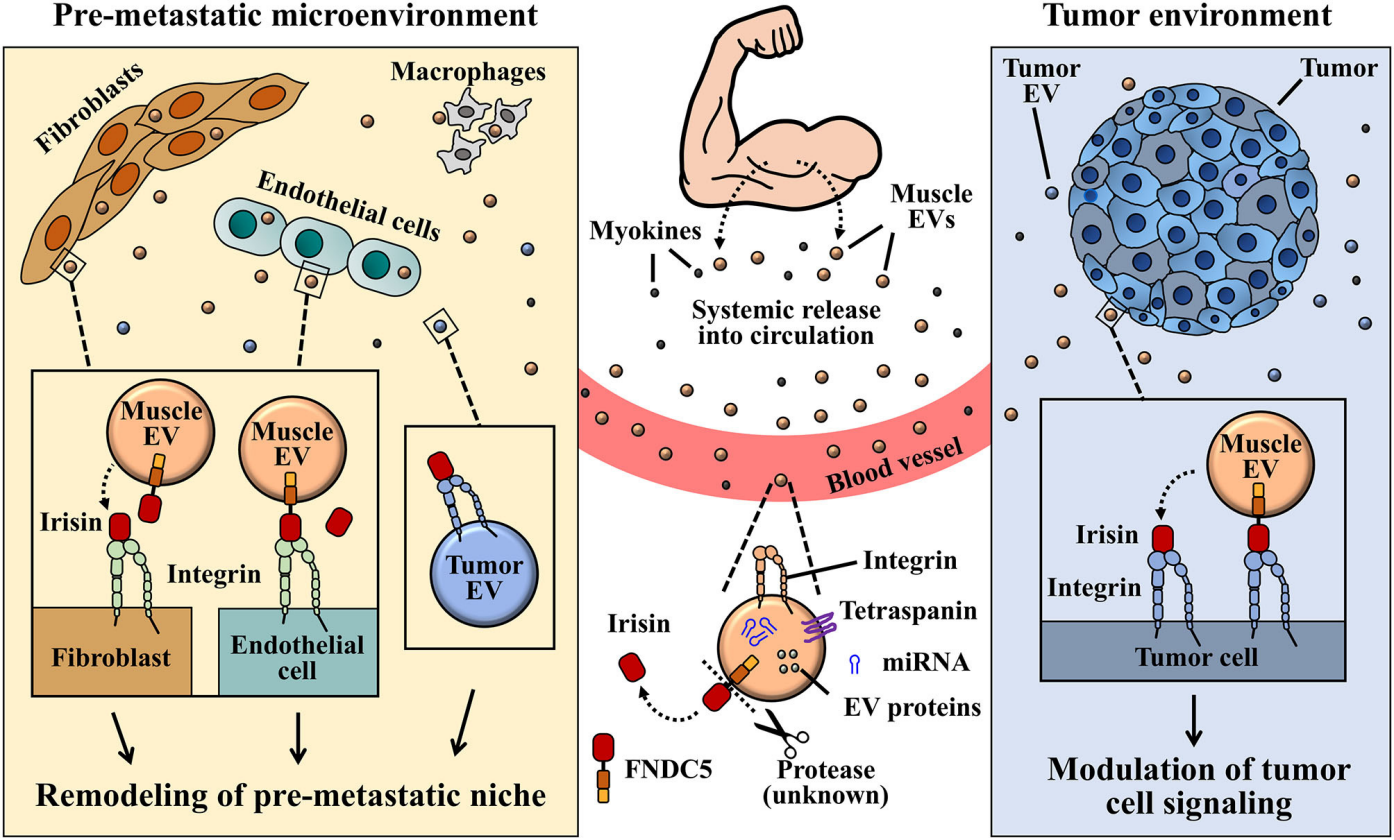
A graphical illustration proposed for the interaction between muscle EV irisin and integrin, and its role in pre-metastatic tumor environment remodeling. Circulatory or EV-bound irisin derived from muscle cells can potentially bind to integrin receptors expressed on several tumor cells or tumor-derived EVs. EVs and irisin released by muscles into circulation, upon reaching pre-metastatic microenvironment, may engage tumor-derived EVs via EV-bound irisin/EV integrin and/or free-irisin/EV integrin interactions. Signaling from integrin-irisin interaction in tumor microenvironment could mediate functional regulation of tumor-derived exosomes in the remodeling of pre-metastatic microenvironment.

Irisin, a recent discovery to the family of adipomyokines (exerting its effect on both adipose and muscle tissues) is a thermogenic protein thought to be vital in energy metabolism (Boström et al., 2012; Rodríguez et al., 2017). Irisin has been proposed to bridge the communication between the muscle and other tissues of the body, and therefore has gained much attention in research relating to metabolism and tissue crosstalk (Pukajło et al., 2015). Irisin is a fragment of the fibronectin type III domain-containing protein 5 (FNDC5) on the cell membrane of myocytes, adipocytes, and other cell types (liver, brain, stomach, etc.) (Aydin et al., 2014). The FNDC5 protein has a cytoplasmic C-terminal portion and an N-terminal extracellular portion that is proteolytically cleaved and released into circulation as irisin (Panati et al., 2018). Physical activity, especially high intensity exercise and resistance training have been shown to increase the levels of irisin in circulation (Tsuchiya et al., 2014, 2015). Although the specific receptor for irisin was yet to be identified until recently, the pivotal study by Spiegelman and colleagues has demonstrated that irisin binds to integrins (Kim H. et al., 2018). Integrin-ligand interactions activate several downstream signaling pathways that regulate cellular processes (Figures 2, 3). We have previously reviewed and discussed the emergence of irisin and integrin-ligand interactions in the context of cancer, metabolic disorders, and inflammation (Park et al., 2020). Irisin binds to several integrins, such as αVβ5 and α5β1 on bone and fat cells, however, binding affinity seems to be higher with the α_V_ family of integrins (Kim H. et al., 2018). Irisin increases the expression of thermogenin (UPC-1, a mitochondrial protein in adipose tissue lipid droplets) in matured fat cells and facilitates browning of white adipose tissue (WAT), leading to the formation of beige-adipose tissue. Although not directly inducing browning, irisin inhibits the formation of new adipose cells, and thus, negatively regulates adipogenesis via irisin-induced phosphorylation of mitogen-activated protein kinases (Zhang et al., 2014; Korta et al., 2019). *In vitro* studies suggest that irisin plays a protective anti-inflammatory role by suppressing the production of pro-inflammatory cytokines in fat cells and immune cells, and therefore alleviates obesity-induced inflammation. Adipocytes are sensitive to irisin and tend to release relatively low levels of IL-6, IL-1β, monocyte chemotactic protein 1 (MCP 1), and TNF-α under the effect of irisin *in vitro*, potentially via the regulation of downstream signaling of Toll-like receptor 4/myeloid differentiation primary response 88 (TLR4/MyD88) (Mazur-Bialy et al., 2017a,b) (Figure 2).

Aside adipocytes, bone cells (osteocytes) have been identified to be sensitive to irisin, and thus, irisin partly plays a role in bone remodeling. Upon irisin treatment, osteocytes under oxidative stress conditions show a reduced ratio of apoptotic cells in culture (Kim H. et al., 2018). Irisin directly regulates osteoclast function and differentiation by stimulating the release of osteoclastogenic promoters like RANKL and sclerostin, even at normal circulatory concentrations (Estell et al., 2020). Earlier studies had also identified osteoblasts as direct targets of irisin; an enhancer of osteoblast differentiation (Colaianni et al., 2014). Although irisin dually targets osteocytes and osteoblasts, the effect of irisin on different bone cell types greatly depends on the circulatory concentrations of the protein, since varying concentrations of irisin treatment have demonstrated contrasting effects in *in vitro* studies (Colaianni et al., 2014; Estell et al., 2020). Further studies in this area are needed to clearly understand the mechanisms by which irisin mediates bone metabolism.

Recently, the involvement of irisin in inflammatory disease has been demonstrated, particularly with intestinal inflammation. Binding of irisin to αVβ5 integrin units on intestinal epithelial cells have been shown to alleviate epithelial barrier dysfunction during gut injury. Irisin restored epithelial barrier function via the activation of αVβ5/AMPK-UCP 2 signaling pathway, while reducing oxidative stress and apoptosis in enterocytes (Bi et al., 2020). Taken together, irisin is a physiologically beneficial adipomyokine that possess properties contributing to oxidative stress alleviation (Bi et al., 2020), anti-inflammation (Mazur-Bialy et al., 2017a,b; Xiong et al., 2018), and anti-metastatic effects (Rabiee et al., 2020). It is worth mentioning that, there are a few conditions in which irisin has been demonstrated to promote inflammation. This is particularly shown in hepatocellular carcinoma (HCC) whereby, the increased hepatic mRNA levels of FNDC5/irisin in HCC patients correlated with increased expression of proinflammatory markers, such as IL-6 and TNF-α. However, mechanisms underlying the irisin-induced inflammation in hepatic carcinoma remains to be completely understood (Gaggini et al., 2017).

## Exosomal Integrin-Irisin Signaling (Potential Implication for Cancer Metastasis)

The discovery of integrins belonging to the αV family as receptors for irisin signaling (Kim H. et al., 2018) has been a great leap toward understanding how irisin could regulate metabolism of both host and tumor cells, considering that, integrins are ubiquitously expressed. Although irisin has not yet been identified as a cargo content of exosomes released by muscle and adipose cells, circulating irisin may engage integrin receptors on exosomes to mediate various niche pre-conditioning (Figure 3). Taking into consideration that exosomes express and transport integrins (identified receptor for irisin) on their surfaces, the potential “cargoing” of irisin in muscle-derived exosomes can be proposed in a few ways. One of such possibilities lies in the endocytosis of surface proteins on muscle cells during EV biogenesis, leading to the formation of MVBs that may package membrane-bound integrin-irisin complexes (Figure 1). This mechanism has been proposed for the exosomal packaging of ECM proteins, such as fibronectin (Sung et al., 2015).

The expression of αV integrins is highly upregulated in several cancers and tends to augment the metastatic phenotype of tumor cells, thus promoting their migration (Teti et al., 2002; McCabe et al., 2007; Haeger et al., 2020). Expression of integrins is not confined to cancer cells only; exosomes obtained from tumors contain a myriad of functional tumor-derived integrins that precondition microenvironments for organ-specific metastasis (Hoshino et al., 2015). Tumor-derived exosomes are capable of transferring exosomal integrins to tumorigenic and non-tumorigenic cells. For example, Fedele et al. demonstrated that αVβ6 which is highly expressed on prostate cancer cells was transferable among different subsets of prostate cancer cell lines via their exosomes. Prostate cancer cell-derived exosomes which expressed functional integrin αVβ6 were taken up by recipient prostate cancer cells that subsequently re-expressed the integrin on their surfaces (Fedele et al., 2015). This group also showed that another member of the αV integrin family, αVβ3, was transferred from tumorigenic to non-tumorigenic prostate epithelial cells via exosomes and induced functional changes like enhanced migration and adhesion in recipient cells (Singh et al., 2016). Other studies confirm the *in vitro* transfer of exosomal αVβ3 integrin to β3-negative cells resulting in the acquisition of ligand binding activity (Krishn et al., 2019). Indeed, integrins expressed on the surface of exosomes are able to bind and adhere to cell surface, or ECM proteins, such as collagen and fibronectin, thus, support integrin binding-mediated cell adhesion (Park et al., 2019b; Altei et al., 2020).

Although the particular role of irisin in cancer progression still remains to be elucidated, serum levels of irisin is markedly altered in several cancers (Zhang et al., 2018). However, there are contrasting reports on the effects of irisin in association with cells. For example, irisin was found to suppress the viability, proliferation and migration of some malignant breast cancer cells (MCF-7 and MDA-MB-231, by way of induced apoptosis) but not others like MCF-10A breast epithelial cells (Gannon et al., 2015). Additionally, irisin signaling has been found to inhibit the proliferation, migration, and invasion of lung cancer (Shao et al., 2017). This effect results from the irisin-induced inhibition of Snail transcription factors via the PI3K/AKT pathway, resulting in the reduced expression of epithelial-mesenchymal transition (EMT) markers (Shao et al., 2017) (Figure 2). Nonetheless, these findings point to the potentially protective and organ specific role played by irisin in cancer progression and metastasis (Zhang et al., 2018).

Taken together, the evidence supporting exosomal carriage of functional and transferable integrin proteins, such as those belonging to the αV family (Fedele et al., 2015; Singh et al., 2016; Krishn et al., 2019), and the identification of integrins as signaling receptors (Kim H. et al., 2018) propose irisin-exosome interactions as potential players in niche regulation especially in cancer metastasis (Figures 1–3). Irisin may negatively impact cancer progression and metastasis indirectly via their interaction with tumor-derived exosomes. Extracellular vesicles designed to precondition distant microenvironment for organotropic metastasis could potentially be altered by the activation of integrin-irisin signaling cascades (Figures 2, 3). Further studies in the area of irisin-exosomal integrin interaction is warranted to ascertain molecular mechanisms that underlie the remodeling of microenvironment and tumor metastasis. Muscle-derived myokines and EVs may hold promising contributions toward therapeutic breakthroughs in cancer management.

## Perspective

With the growing evidence of their involvement in the autocrine/paracrine/endocrine regulation of metabolic pathways (Seldin and Wong, 2012) and inflammation (Febbraio and Pedersen, 2005), muscle cells and muscle cell-derived EVs deserve attention in our quest to demystify pathophysiological processes underlying various health condition, including cancers. Muscle-derived EVs that cargo myokines and other biologic modulators (nucleic acids, growth factors, etc.) could potentially modulate the remodeling of niches in vital sites, such as liver and adipose tissues. The anti-inflammatory effect of skeletal muscle activity mediated by myokines (e.g., IL-6) could negatively contribute to the establishment of favorable inflammatory niche conditions, necessary for cancer metastasis. Although more studies to support exosomal packaging and release of important myokines like irisin, IL-6, and myonectin are desired, the growing evidence of muscle EV secretion and remarkable encapsulation of muscle-specific biologic contents (transferable to various cell types and also implicated in cancer progression) (Singh et al., 2016; Wang et al., 2016; Li et al., 2018; Xu X. et al., 2018; Krishn et al., 2019) provide substantial cues for further investigations. Metabolic syndrome (including obesity, hyperlipidemia and diabetes) burden organs with pro-tumorigenic and metastatic cascades associated with chronic inflammation, increased oxidative stress, and deregulated cellular signals (Harvey et al., 2011), and therefore, potentially render tissues niche-ready for cancer metastasis. On one end, because muscle and muscle-derived EVs pack mediators that restitute the effects of metabolic dysregulation, their indirect attribution to the regulation of microenvironment remodeling and the establishment of pre-metastatic homing niches can be hypothesized. Little evidence exists to explain the relationship between muscle-derived EVs and tumor metastasis, however, findings from other pathologies, such as inflammation and metabolic disorders discussed in this review point out a plausible connection that may potentially benefit homeostatic regulation of homing niches against tumor progression metastasis. Further studies are imperative to better understand the role of muscle-derived exosomes in regulating the events of pre-metastatic niches and cancer progression. This may provide novel insights to the development of therapeutic interventions for the management of cancers.

## Author Contributions

SD and MS contributed to the conceptualization, scope, and outline of this review. SD, EJP, PKM, AI, MGA, GO, EK, and MS analyzed the referenced manuscripts in this manuscript and participated in preparing the manuscript. All authors read and approved the final version.

## Conflict of Interest

The authors declare that the research was conducted in the absence of any commercial or financial relationships that could be construed as a potential conflict of interest.
